# A little help from my friends: caring for premature babies in a war zone

**DOI:** 10.1186/1746-4358-2-3

**Published:** 2007-02-03

**Authors:** Heather Harris

**Affiliations:** 1 Birralee Maternity Unit, Box Hill Hospital, Box Hill, Australia; 2 Formerly, Médecins Sans Frontières, Cote d'Ivoire, West Africa

## Abstract

This paper is a narrative of some aspects of my work as a midwife with Médecins Sans Frontieres (MSF) in West Africa. I was situated in an isolated north-western regional hospital in an area under rebel military control in 2004–2005 in the Côte d'Ivoire during the civil war which divides the north and south of the country. Access to health care is severely curtailed in this politically unstable environment resulting in much avoidable illness including many premature births. It is a short account of methods used to care for premature babies in a resource poor setting. Equipment was basic, necessitating a creative use of available resources. Providing warmth, oxygen and adequate feeding were often sufficient for a successful outcome for many premature babies. This paper is a combination of descriptions of health care interspersed with case studies.

## Review

Côte d'Ivoire is in West Africa and has a population of over 17 million. It was a former French colony so the official language of the educated population is French though many indigenous languages are spoken in the villages away from the main cities. This beautiful country has been gripped by civil war since late 2002, with one result the collapse of the health care system particularly in the northern and western region, which is effectively cut off from government control. The World Health Organization (WHO) lists Côte d'Ivoire as 79/95 on the Human Poverty Index scale with 15.5% earn less than $US 1 a day [1]. The Gross National Per Capita Income is estimated at US$770 per annum [2]. Life expectancy for men is 37.6 years and for women 41.3 years [1]. Skilled birth attendants assist with 62.5% of births (2000) compared with 99.3% in Australia. The Infant Mortality Rate is 118/1000 of live births [2], while in Australia it is 5/1000 [3]. Child mortality rates for children under 5 is even higher – 162/1000 for girls and 225/100 for boys [1]. Breastfeeding is considered the global "gold standard" for all babies less than six months of age, but in a poverty-stricken, isolated setting with few reliable health structures in place, it can be a stark choice between life and death.

Almost all births occurred in the villages with the more difficult births brought to the hospital. These resilient people rarely seek medical assistance unless they are extremely ill. Treatment with indigenous medication was their first recourse and if that failed, they would present for western medical care.

The author spent much of 2004 and 2005 working as a midwife with Médecins Sans Frontières (MSF) in the far west of the country which is under the control of the breakaway rebel group, the *Forces Nouvelles*. MSF provided primary health care at a rural hospital in Danané and from daily mobile clinics. Most of the population lives in small villages scattered throughout the bush. The mobile clinics went out every day to these isolated sites to give care to those who made their way to the clinic. During the wet season tracks are often difficult to negotiate when parts of the road or makeshift bridges are washed away. Most people walk, use a pushbike, take a bush taxi if they can afford it or hitch a ride on a passing truck. It was not unusual to see very ill people being wheeled into the clinics in a wheelbarrow or being carried on someone's back.

Falciparum malaria is endemic in this area and was a big killer of children and often resulted in premature births in infected pregnant women. Almost half of the pregnant women seen in the clinics tested positive for malaria and many others suffered concurrent opportunistic infections including STDs (sexually transmitted diseases) and were often chronically anaemic. Malaria was detected using a simple blood test, but most other infections were diagnosed symptomatically and treated with relevant antibiotics. Many primigravidas were in their early teens and it was not unusual for a 30 year old to be carrying her tenth pregnancy. We had access to corticosteroids for pregnant women who arrived with ruptured membranes if they were less than 34 weeks gestation or in threatened premature labour. Whenever this was given, invariably the baby survived if labour could not be stopped using salbutamol. It is difficult to know the numbers of births in the countryside as not all are registered. However of the 160 or more births per month at the hospital – caesarian rate of 15 – 17% – at least six or more per month who survived their birth were significantly premature – weighing less than 1500 grams, with a number around 1000 grams or less. This number is only those women and babies who presented to the hospital for care. It is unknown how many premature babies or other pregnancies were dealt with in the villages, nor the outcomes but if we look at the national mortality rates, it is reasonable to assume there are many deaths of both mothers and babies which are never recorded. Multiple pregnancies are very common and in the time the author worked there, three sets of triplets and a set of quadruplets were born at the hospital, all of whom survived their birth, and a number who went home alive.

The survivors of premature births became known as "*Les Petites Princesses*" as almost without exception they were girls. (See Case study 1. Initiating feeding regime for premature baby).

### Case study 1. Initiating feeding regime for premature baby

A tiny bundle arrived swathed in a colourful cloth wrap one morning. The baby girl inside weighed 800 grams and was so cold her temperature did not register. But she was pink, active and had a little rib retraction. We warmed her by wrapping her in a foil blanket and surrounding her with gloves filled with warm water. We inserted an IV (intravenous line) and her 15 year old mother was encouraged to express breast milk every few hours. We fed it via the baby's naso-gastric tube every two hours, starting off with 1 or 2 mls and increasing it as supply and her tolerance increased. Oxygen and intravenous antibiotics were started as her lungs were crackly and infection was a strong possibility. She was the first of our "*Petites Princesses*" and was crowned "Queen of the Maternity" when she reached 1000 grams. She went home weighing 1500 grams, tied securely onto her mother's back, cuddled safely into the base of her spine and a perky cap on her tight black curls. (One of the most precious gifts received by the author is a t-shirt with "*Mere des Princesses*" printed across the front.)

Most premature babies who survived their birth were around 29 to 33 weeks gestation based upon clinical assessment. When I arrived in Danané there was little effort to resuscitate them as the general expectation was that they would die. However as we adopted a more proactive approach, the survival rate increased. As staff became more empowered their efforts increased accordingly, sometimes to heroic proportions. With robust resuscitation, prompt provision of oxygen and efforts to maintain body temperature these babies began to survive.

### Kangaroo care

Thermoregulation was a major challenge. Côte d'Ivoire is a tropical country and swaddling newborn babies is not a routine practice. It is normal for babies to be only loosely wrapped at birth, allowing significant cooling. For the robust term baby it was never a great problem, but for premature babies, it was critical. Each premature baby was wrapped from birth in a gold foil "space" blanket, readily available from MSF – it is similar to aluminium foil and after wrapping, these babies looked like a small, shiny Christmas present. Even so, it was difficult to maintain their temperature until we initiated kangaroo care. At first the staff, mother and attendant family were extremely skeptical about this and needed much reassurance that it would do no harm. When it was demonstrated how dramatically baby's temperature could be raised with skin to skin care (1°C in 1 hour) staff became enthusiastic. (See Case study 2. Thermoregulation of newborn.) Women reluctant to adopt kangaroo care would often remove their baby after 30 – 60 minutes and leave it wrapped on the bed beside them. Eventually they were persuaded to do K. Care in 2 – 3 daily "treatments."

### Case study 2. Thermoregulation of newborn

One of the nursing aides, Diakite, is concerned about a premature baby who has been admitted after a village birth. The little boy's temperature is unrecordable and he weighs less than 1000 grams. With great deliberation, Diakite removes his shirt, lays the tiny boy spread-eagled across his chest, and then covers him with a bright towel. They both lie back on the sheetless, vinyl covered ward bed, Diakite grinning broadly and within 15 minutes the grunting respirations of the baby have receded and he lies sleeping peacefully as his body warms. We insert a naso-gastric (NG) tube and oxygen flows from the air concentrator into his large face mask. The first precious mls of colostrum are expressed from the unconscious eclamptic mother and are fed down his NG tube. With magnesium sulphate treatment, the mother regained consciousness next day, was able to resume care of her baby and both eventually went home after some weeks.

Sometimes surgical gloves filled with warm water were placed around the baby. In a busy ward of twelve mothers and babies and only one nursing aide to give all their care, this was very time consuming and not workable. Finally large hard plastic water filled bricks normally frozen and used for the maintenance of the cold chain were used. Instead of freezing, I immersed them in very hot water until warm, then laid cotton wool over them, placed the baby on top of that and all were wrapped together. It worked very well and keeping premature and sick babies warm when kangaroo care was not possible became a far less difficult task. Flies were a constant hazard so babies' faces were protected by draping a piece of dressing gauze across them or often the whole baby was cocooned in a bright cotton wrap so that finding them was like carefully unwrapping a precious parcel. There is a fly called the Tumbu fly which lays its eggs on exposed skin and a daily inspection was necessary to ensure they were not playing host to larvae.

### Stabilising and feeding premature babies

This was the next challenge. The objective was to avoid/treat initial hypoglycaemia, maintain hydration, minimize weight loss and ensure weight gain. After birth most babies were cannulated as soon as possible (see Case study 3. Initial stabilization) as the possibility of infection requiring antibiotics was high. Also many were too small to take oral feeds and we had to keep them hydrated parenterally.

### Case study 3. Initial stabilization

A premature baby needs an IV. I don't have the skills and nor do any of the staff on today. We don't have any intra-osseous needles. I radio for Jayeo, a very large, highly experienced young ex-Benin Army nurse. He arrives, prepares everything and ties the rim of a glove around the minute arm as a tourniquet. His big hands expertly search for a tiny vein to cannulate in an arm the diameter of a biro barrel. He is an expert and invariably finds a vein. Slowly he inserts the cannula, easing it gently along the hair-thin vein and checking it is patent by tenderly flushing with normal saline. After each success he exclaims triumphantly "*Aha c'est ca Edda*!" claps his hands and then fashions a tiny arm board out of half a wooden tongue depressor covered in cotton wool. Minutes later, all is done, arm is stable and we can start fluids.

Breast expression was started immediately when baby was too small to suckle and expressed breast milk given two hourly via the naso-gastric (NG) tube. Expressing breast milk is not a normal activity for these mothers and like many women in the industrialized world many found it confusing and difficult to master initially. However with encouragement small amounts were extracted and stored beside the mother's bed. There was no refrigeration available but as the colostrum was used within a short time this was not an issue.

I often considered milk banking, but there were no refrigeration or sterilization facilities so it remained only a dream. If there was insufficient expressed breast milk, 10% dextrose was given orally until the milk came in. Premature infant formula was unavailable and in fact infant formula was never used as we wanted the baby to be fed human milk exclusively to safeguard its health. If we had used infant formula, it may have given a message that formula is safe, that there is no need to try hard with breastfeeding as a large international non-government organization (INGO) will supply formula and that this was acceptable and even preferable to mother's milk.

Intravenous antibiotics were commenced if there were any signs of infection (usually respiratory) and IV dextrose 10% if baby was unable to tolerate oral feeding. This was combined with Ringers Solution to achieve some electrolyte balance. Staffing levels were minimal and skill levels not as high as trained nursing staff, so much supervision was needed in the early days to avoid the potential danger of regurgitation and inhalation of gastric contents when feeding very low birth weight babies early with breast milk. Alternatively, breast milk was a more balanced nutrition and I tried to initiate it as soon as was practicable. Infant multivitamin drops although scarce, were used when available and appeared to improve the infant's condition markedly. Staff instructions were simple – "Keep the baby warm, pink and sweet, that's all you have to do."

### Maintaining oxygenation

For premature babies requiring extra oxygen there was an air concentrator and some bottled oxygen available. The concentrator extracts oxygen from the ambient air and delivers it via a mask, giving a limitless supply. It is a machine often used in resource poor settings. It depends upon electricity to function, and if there was an electrical blackout – not uncommon – oxygen was then given from the precious cylinders. Even the smallest infant oxygen masks were too big, covering the entire little face from forehead to chin, so a piece of gauze was packed around the edges to stop the oxygen escaping. No oxygen head box or nasal prongs were available.

### Feeding methods

If they survived the first 4 to 5 days, most premature babies were able to breastfeed very early. My midwifery training had always emphasised this was not possible but the reality is very different. Many were partially breastfeeding at 1100 to 1200 grams and certainly by 1400 to 1500 grams were fully breastfed. We enabled maximum milk transfer at the breast very early by teaching mothers breast compression during a breast feed. As the mother squeezed and held her breast, she pushed a bolus of milk down to the nipple, where it dripped into the baby's mouth and encouraged another surge of suck/swallowing activity. This simple form of assisted breastfeeding was enthusiastically adopted once the mother saw how easy and effective it was.

Expressed breast milk was given either by cup or finger feeding. There were very few receptacles small enough to use as a cup so usually we finger fed. This involved the mother putting her clean little finger into the baby's mouth, pad uppermost touching the palate to stimulate a sucking reflex, waiting for the baby to start sucking and then staff slowly giving milk via a syringe nozzle inserted beside the finger. Finger feeding is a highly effective, simple and mother-inclusive way to feed. All mothers and staff who learned this method were fascinated to watch how well it worked and haw effective it was. There was not a bottle or teat in the hospital as we did not want to model a western form of infant feeding in an environment where it is virtually impossible to do safely.

The average hospital stay for premature babies was around three weeks and most went home weighing around 1700 – 1800 grams. Many of the mothers of these premature babies had other small children back in their village and the longer they were absent, the more potentially precarious their other children's survival became. It was a juggling act for these women, trying to balance the needs of this tiny one against the needs of their other offspring. Other family members assumed responsibility for the children's welfare in the mother's absence, but it put an added strain upon the often meagre resources available to these families.

The local method for increasing milk supply is to massage the breasts with a sugar mixture which leaves a shiny sticky film on the skin. It was a very common remedy although I am unsure of the basis for its use. I could never really see any increase in lactogenesis from this method but it was used frequently. Access to galactogues such as metoclopramide was possible from the hospital pharmacy. This was given as a 10 mg tablet 3 times a day for 7 days then slowly reduced as milk supply increased. There was no domperidone in the pharmacy and using metoclopramide was a previously unheard of way to stimulate milk production there. It was usually successful if the mother was basically well, but those who were already in poor health understandably did not increase milk production particularly well. Two meals a day were provided at the hospital for all patients, though often not eaten as it was food donated from World Food Program and was not their usual fare, so was not always popular. Every effort was made to enable the mother to rest as much as possible. There was infant formula in the hospital pharmacy but very rarely prescribed. We even had some "follow-on" formula which had arrived from somewhere and which was never used, so to prevent it being wasted by going out of date, I sent it off to the kitchen to be included in the general cooking. The cost of a can of formula in the town pharmacy was more than a day's wages. In this setting infant formula is a death sentence and if our efforts to increase supply were not successful, a message would be sent to the family to find someone else to donate breastmilk or arrange a wet-nurse. Sometimes this was easy, at other times, very difficult. This was due at times to reluctance to suckle a baby which was not "family" or because the breastfeeding mother was afraid that she could not supply enough for two, or that her own baby would be disadvantaged or that she may fall ill herself or that the family could not afford the cost of hiring a wet-nurse. When one was found, there was always the chance they may suddenly leave the village to travel elsewhere and the baby again had no-one to feed it. A number of grandmothers I met were breastfeeding their orphaned grandchild. (See Case study 4: Feeding multiples.)

### Case study 4: Feeding multiples

A young mother arrived one day accompanied by a female relative. Each carried two babies – one each tied on their backs and another balanced on their hips. Three babies were 3 months old and the largest was 5 months old. This courageous young woman had adopted her sister's triplets after their mother had died. I never could find out why she died as no-one seemed to know, but orphans were quite common. All these babies were thriving at this stage but she was having difficulty supplying sufficient breast milk for all of them. We initiated a system where she came every month and we weighed her babies and gave her enough infant formula to supplement the children. At the time I left the project, all babies were alive and well.

## Conclusion

The survival of many of these very small babies is a tribute to the dedication of the national staff – particularly in a setting where there is virtually no technology and survival depends on close monitoring, commitment and innovative thinking. All staff are "untrained" but are intelligent, caring, responsible and highly skilled at their work. There are many "princesses" alive today who would not be here without the care of these wonderful men and women. In conclusion, the care of these babies and their mothers was challenging but also exhilarating whenever there was a successful outcome. I learned much about premature baby care in this environment and it is always amazing to see how resilient very premature babies can be when faced with what may at first appear to be insurmountable obstacles.

## Competing interests

The author(s) declare that they have no competing interests.
